# Failure to improve task performance after visuomotor training with error reduction feedback for young adults

**DOI:** 10.3389/fphys.2023.1066325

**Published:** 2023-03-08

**Authors:** Yen-Ting Lin, Yi-Ching Chen, Gwo-Ching Chang, Ing-Shiou Hwang

**Affiliations:** ^1^ Department of Ball Sport, National Taiwan University of Sport, Taichung City, Taiwan; ^2^ Department of Physical Therapy, College of Medical Science and Technology, Chung Shan Medical University, Taichung City, Taiwan; ^3^ Physical Therapy Room, Chung Shan Medical University Hospital, Taichung City, Taiwan; ^4^ Department of Information Engineering, I-Shou University, Kaohsiung City, Taiwan; ^5^ Department of Physical Therapy, College of Medicine, National Cheng Kung University, Tainan City, Taiwan; ^6^ Institute of Allied Health Sciences, College of Medicine, National Cheng Kung University, Tainan City, Taiwan

**Keywords:** EMG, motor control, motor units, visuomotor integration, error

## Abstract

Visual feedback that reinforces accurate movements may motivate skill acquisition by promoting self-confidence. This study investigated neuromuscular adaptations to visuomotor training with visual feedback with virtual error reduction. Twenty-eight young adults (24.6 ± 1.6 years) were assigned to error reduction (ER) (n = 14) and control (n = 14) groups to train on a bi-rhythmic force task. The ER group received visual feedback and the displayed errors were 50% of the real errors in size. The control group was trained with visual feedback with no reduction in errors. Training-related differences in task accuracy, force behaviors, and motor unit discharge were contrasted between the two groups. The tracking error of the control group progressively declined, whereas the tracking error of the ER group was not evidently reduced in the practice sessions. In the post-test, only the control group exhibited significant task improvements with smaller error size (*p* = .015) and force enhancement at the target frequencies (*p* = .001). The motor unit discharge of the control group was training-modulated, as indicated by a reduction of the mean inter-spike interval (*p* = .018) and smaller low-frequency discharge fluctuations (*p* = .017) with enhanced firing at the target frequencies of the force task (*p* = .002). In contrast, the ER group showed no training-related modulation of motor unit behaviors. In conclusion, for young adults, ER feedback does not induce neuromuscular adaptations to the trained visuomotor task, which is conceptually attributable to intrinsic error dead-zones.

## 1 Introduction

A visuomotor task requires the coupling of visual targets to effector motor behaviors with error-based negative feedback (Taylor et al., 2014). Visual feedback sensitivity could affect effector motor behaviors, including changes in common synaptic inputs and discharge patterns of spinal motor neurons (Laine et al., 2014; Farina et al., 2017 ). In addition to the error-based negative feedback, a reward-based action-selection process can reinforce positive aspects of movement execution to advance motor learning. The basal ganglia (Kim et al., 2017) and parietal cortex (Mutha et al., 2011) are primarily involved in reward processing. Researchers have shown that errorless practice in the early stage of skill acquisition fosters implicit learning (White et al., 2014; Kal et al., 2018), and motor skills are more resistant to degradation in a pressure situation (Lee et al., 2016). There is a critical balance of error making and error avoidance to optimize motor learning (Kelley, 1969; Krakauer and Mazzoni, 2011; Lee et al., 2016).

Variations in the error size of feedback can develop a new calibration process for performance adjustments. Regardless of the feedback form, virtually signaling a worse outcome (or a greater error size) than the real performance is known as error amplification (EA) feedback (Kao et al., 2013; Abdollahi et al., 2014; Chen et al., 2017a). EA could tax the limited attention resources for processing exaggerated error information (Milanese et al., 2016; Marchal-Crespo et al., 2017; Milanese et al., 2017). In contrast, error reduction (ER) feedback displays smaller execution errors than actual performance feedback does (Hwang et al., 2017; Hwang et al., 2020). ER is associated with some behavioral advantages: It positively reinforces task success and reduces stress from committing errors, such as by enhancing motivation and self-efficacy during task completion (Hwang et al., 2020). Conceptually, ER is a trade-off between the error-based adaptation and reward-based action-selection processes. Central control of motor units in a static force task could vary with the use of ER; such variation would manifest as the less coherent discharge of motor units with higher recruitment thresholds (Hwang et al., 2017). Although ER seems to temporarily undermine task performance in skilled subjects (Hwang et al., 2017), it might improve task performance by reducing performance stress during the early stages of skill acquisition (Ong et al., 2010).

To our knowledge, no previous studies have investigated the effect of visuomotor training with ER for young adults from the aspects of force dynamics and motor unit behaviors. For virtual minimization of execution errors, ER could reinforce correct schemas for less performance stress in the initial skill acquisition (Ong et al., 2010; Hwang et al., 2020). Hence, the subjects could be better motivated and thus achieve a superior learning effect after training (Chiviacowsky and Wulf, 2007; Saemi et al., 2012). For potential perception-induced motor facilitation, the purpose of this study was to contrast behavioral and neurophysiological mechanisms underlying short-term training with and without ER for a bi-rhythmic force task. Namely, the participants learned to couple a force trajectory to a target signal of combined sinusoidal waves of two different frequencies, with half of participants receiving ER feedback. We hypothesized that ER training would result in a better performance than would training with feedback with no reduction in errors, based on our previous results that showed potential immediate effect of visual ER for older adults (Hwang et al., 2020). Regarding the potential benefit of visual ER, ER training was hypothesized to increase coherent discharge at the target frequencies and reduce the discharge variability of motor units for young adults during a bi-rhythmic force task.

## 2 Methods

### 2.1 Participants

Twenty-eight healthy adults were randomly assigned to one of two groups: an error reduction (ER) group (n = 14; 7 males, 9 females, mean age ±SD = 24.6 ± 1.6 years) and a control group (n = 14; 8 males, 8 females, mean age ±SD = 25.2 ± 1.8 years). They were self-reported to be right-hand dominant without known history of neuromuscular disease. The subjects visited the laboratory on 3 occasions (pre-test, training visits, and post-test) scheduled on three successive days. The study was approved by the Chung-Shan Medical University Hospital Institutional Review Board (No.CS2-16072), and all subjects provided written informed consent for the experiment.

### 2.2 Experimental design and experimental system

**FIGURE 1:**
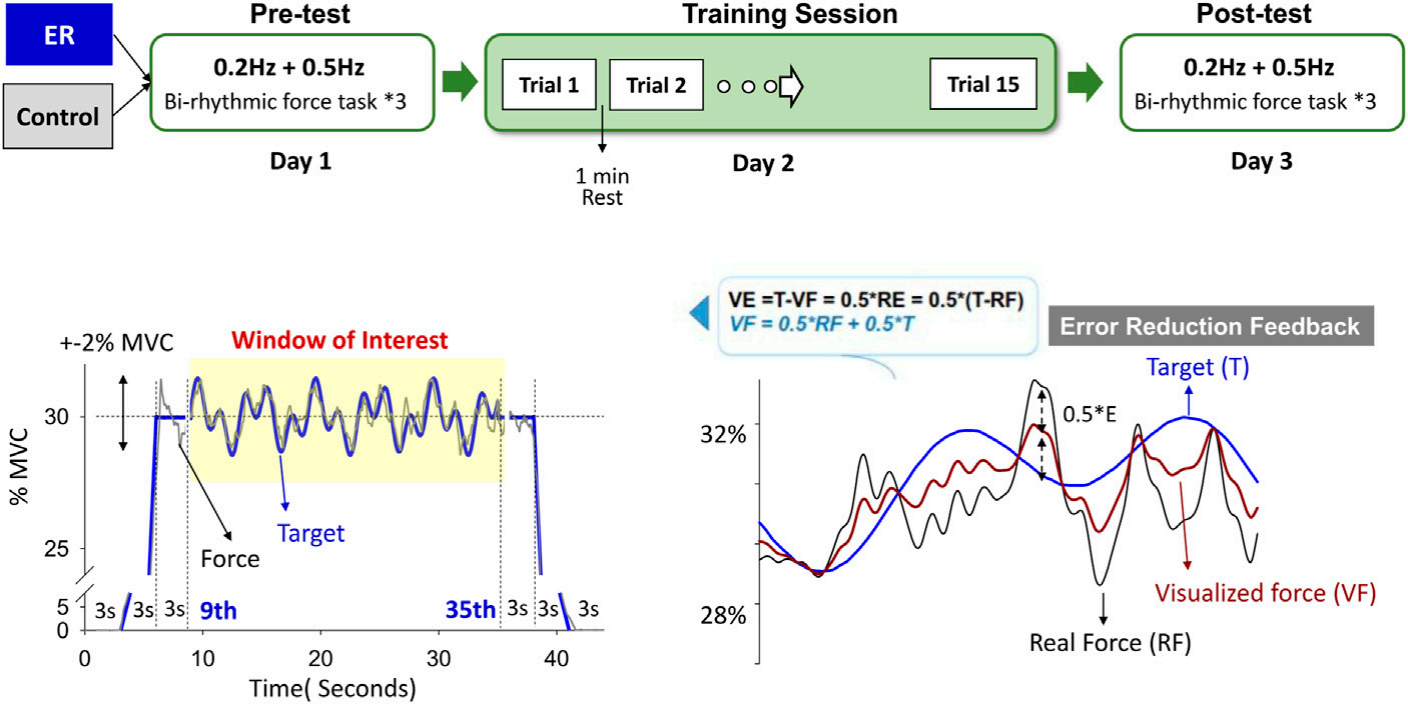
Experimental training protocol. The pre-test (Day1) and post-test (Day 3) consisted of a bi-rhythmic force task to contrast the short-term training effect using feedback with no reduction in errors (control) and error reduction (ER) feedback. The bi-rhythmic task required the subjects to track a visual target that combined sinusoidal waves of 0.2 Hz and 0.5 Hz fluctuating around 30% ± 2% MVC (subplot in the lower left). With simple mathematical transformation (subplot in the lower right), the subject perceived errors reduced by 50% with error reduction feedback during training. The force output displayed on the monitor (visualized force, VF) was equal to the sum of half of the real force (RF) plus half of the target signal (T) (VF = 0.5*(RF + T)). Hence, the size of the visualized tracking error was half of the real error (RE) of the force-tracking task (VE = T-VF = T-0.5*(RF + T) = 0.5*(T-RF) = 0.5*RE). The training task in Day 2 was the same bi-rhythmic force task.

This study used a randomized, repeated measures, between-groups, parallel design. The participants visited the laboratory 3 times at 1-day intervals (Figure 1). During the first visit for the pre-test (Day 1), maximal voluntary contraction (MVC) of isometric index abduction was defined as the highest value of three successive MVC tests of 3 s, which were separated by 3-min rest periods. Three trials of a bi-rhythmic force task under visual feedback were assessed during the pre-test visit. The participants were seated 60 cm in front of a computer monitor, with the forearm of the dominant limb resting and restrained on a thermoplastic splint. They exerted the isometric force by index abduction of the dominant hand to couple a target signal on a computer screen. The target signal comprised a 3-s latent period, a 3-s ramp-up/ramp-down phase to 30% MVC, and 3 s of the static level of 30% MVC at the beginning and end of the contraction. In the window of interest (9^th^–35th seconds), the bi-rhythmic force tasks required the participants to exert combined sinusoidal forces of 0.2 Hz and 0.5 Hz that fluctuated around 30% ± 2% MVC (Figure 1, lower left). Each contraction trial was 44 s. Rest periods of at least 2 min were allowed between the experimental trials in the pre-test. The particular forms of contraction were designed for EMG decomposition using a previous proof-of-algorithm. In addition, the bi-rhythmic force task was expected to add task difficulty and performance stress, unlike the static and mono-rhythmic force tasks used in the previous studies, which manipulated the error size in the visual feedback (Chen et al., 2017a; Chen et al., 2017b; Chen et al., 2017c; Hwang et al., 2020). On Day 2, the practice session contained 15 contraction trials of the same task scheme used on Day 1. Rest periods of 1 min were allowed between training trials. During the final visit for the post-test (Day 3), the participants completed three trials of the bi-rhythmic force task identical to the pre-test.

The subjects in the ER and control groups received different forms of visual feedback when they practiced the bi-rhythmic force tasks. Subjects in the ER group received feedback that was reduced to half the size of the no reduction in errors during training. The force output displayed on the monitor (visualized force, VF) was equal to the sum of half of the real force (RF) plus half of the target signal (T) (VF = 0.5*RF + 0.5*T) (Chen et al., 2017b; Hwang et al., 2017). The size of the visualized tracking error (VE) was reduced so that the participant saw only half of the real error (RE) of the force-tracking task (VE = 0.5*RE) (Figure 1, lower right). The control group was trained with visual feedback that guided force-tracking with feedback with no reduction in errors. On Day 1 and Day 3, the ER and control groups performed bi-rhythmic force-tracking visually guided with feedback with no reduction in errors in the pre-test and post-test.

A force transducer (Model: MB-100, Interface Inc., United States of America) was used to measure the force at the dominant index finger. The force signal was sampled at 1 kHz with an analog-to-digital converter (model USB6251; National Instruments, Austin, United States of America) controlled by a custom program on a LabVIEW platform (LabVIEW v.8.5, National Instruments Inc., Austin, TX, United States of America). A multi-electrode surface EMG with 5 surface pin sensors (0.5 mm diameter) at the corners of a 5 × 5 mm square (Bagnoli sEMG system, Delsys Inc., United States of America) was synchronized to detect the muscle activity of the first dorsal interosseous (FDI) muscle. The electrode position in the pre-test was replicated at every visit to confirm consistent electrode placement in the pre-test and post-test. The EMG signals from each pin sensor were amplified (gain = 1,000) and band-pass filtered (cut-off frequencies of 20 Hz and 450 Hz), followed by a digitization process at a rate of 20 kHz (De Luca et al., 2015a). EMG data were recorded with EMG works v.4.1 (Delsys Inc., United States of America).

### 2.3 Data analysis and signal processing

The force signal was first low-pass filtered (cut-off frequency: 6 Hz) to preclude high-frequency noises and center on the effects of visuo-motor processes on force outputs in the 0–4 Hz band. Only the force output in the window of interest (9^th^–35th seconds) was analyzed. For the pre-test, training session, and post-test, task error in the time domain was denoted as the root mean square (RMS) of the mismatch between the target signal and filtered force output (Err_RMS_). During the practice session, the task errors of the 1st–15th trials were standardized with task errors of the first trial (or normalized task error). Estimated with the FFT-based Welch method (segment length: 3.25 s, overlapping time segment: 25% window length), task accuracy in the spectral domain was represented with a summated peak amplitude of the target frequencies (0.2 Hz and 0.5 Hz) (P_0.2+0.5Hz_) in the amplitude-normalized power spectra of the tracking force. The spectral resolution was 0.002 Hz.

**FIGURE 2:**
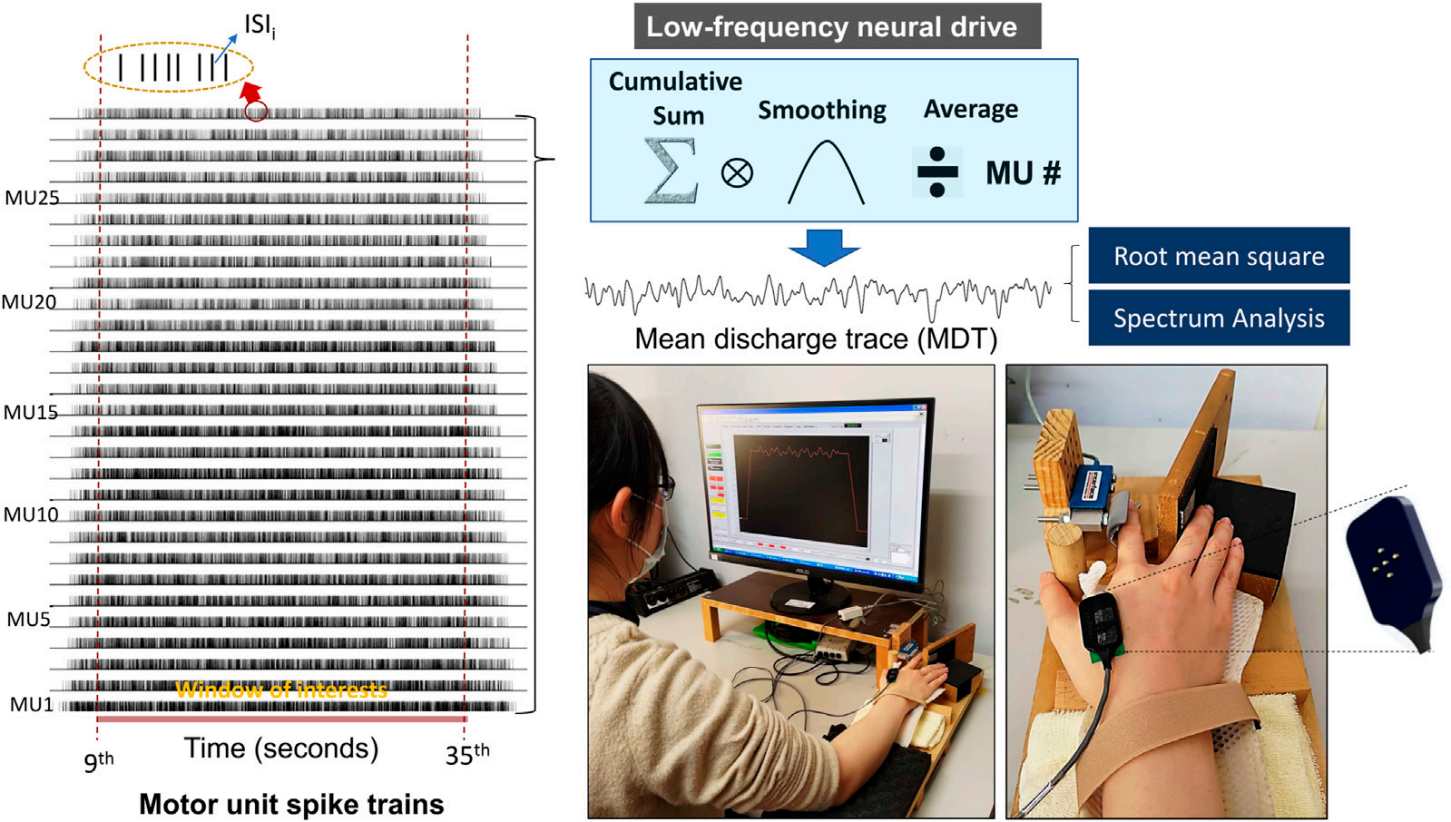
Decomposition results of surface electromyography (EMG) and acquisition of mean discharge trace (MDT) from the cumulative spike train. The smoothing process was performed by convolution of the cumulative spike trains of all identifiable motor units with a Hanning window of 400 ms in duration. Root mean square and spectrum analysis were applied to the MDT in the pre-test and post-test.

With the Precision Decomposition III algorithm (version 1.1, Delsys, Inc., Natick, MA, United States of America) (De Luca et al., 2006; Nawab et al., 2010), multi-channel surface EMG signals were decomposed to the action potential “templates” of motor unit action potential trains (MUAPTs). The decomposition algorithm is reported to reliably detect motor unit activities during rhythmic contractions, rendering motor unit (MU) spike trains having values of 0 or 1 (De Luca et al., 2015a). Only MUs with decomposition accuracy rates higher than 90% were further analyzed (De Luca et al., 2006; Nawab et al., 2010), according to the results of the Decomposition–Synthesis–Decomposition–Compare (DSDC) test. The discharge variables of the motor units in the time window of interest were determined from the whole decomposed EMG data. ISI_mean_ was the mean value of all inter-spike intervals for an individual MUAPT, and M-ISI_GAV_ was the averaged value of the ISI_mean_ for all motor units. The temporal variability of a single MU was determined using the coefficient of variation of ISI (CV-ISI) in one MUAPT, and CV-ISI_GAV_ was the mean value of CV-ISI for all MUs in an experimental trial. The cumulative discharge rate was characterized by the convolution of the cumulative spike trains of properly-identified motor units with a Hanning window of 400 ms (Figure 2). The cumulative discharge rate was divided by the number of properly-identified MUs in an experimental trial to obtain the mean discharge trace (MDT), which highlights the low-frequency neural drive to a muscle by suppressing independent synaptic noises (Farina and Negro, 2015; Farina et al., 2016; Hwang et al., 2019). The power spectrum of the MDT was estimated with a fast Fourier transform and the same Welch method with the same parametric settings was used for the force data. The summated peak amplitudes of target frequencies at 0.2 Hz and 0.5 Hz (P_0.2+0.5 Hz_) in the MDT were also determined.

### 2.4 Statistics

Hotelling’s T-squared statistics were used to examine the group differences (ER vs control) in behavioral performances, including the task accuracy (Err_RMS_ and P_0.2+0.5 Hz_ of force output). Hotelling’s T-squared statistics were also used to examine the group differences (ER vs control) in the discharge variables of individual motor units (M-ISI_GAV_ and CV-ISI_GAV_) and the characteristics of the mean discharge trace (RMS and P_0.2+0.5 Hz_). The post-hoc test was the Simes test, which would not produce over-correction with the Bonferroni test. For all post-hoc hypotheses ( $H=\cap_{i=1}^{m}$ ), the Simes test did not reject elementary *H*_*i*_ if *p*_*i*_ ≤ i*0.05/m for ordered unadjusted *p* values (*p*_*1*_ ≤. ≤ *p*_*m*_). The type 1 error rate using the Simes test proved to be exactly 0.05. During the practice sessions, the paired *t*-test was used to contrast absolute task error of the last trial (the 15th trial) with that of the first trial for both groups. The effect size was determined by partial eta squared (*η*_*p*_^*2*^). All statistical analyses were performed in IBM SPSS Statistics (v19). The level of significance was 0.05.

## 3 Results

### 3.1 Task performance

**FIGURE 3:**
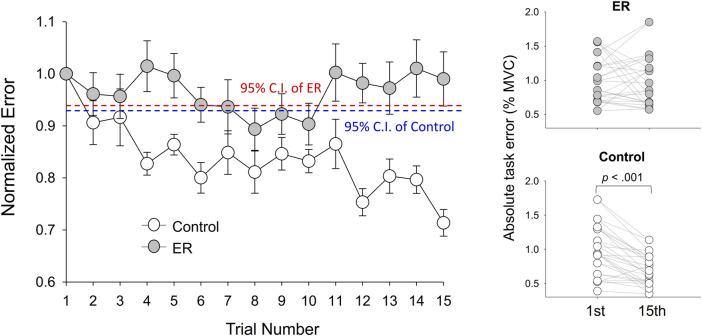
Evolutional changes in normalized task errors during the training session for the ER and control groups. The control group showed a decreasing trend of normalized errors across training sessions. Absolute task error was denoted as the RMS value of the mismatches between the target and tracking force (% MVC). (95% CI: 95% confidence interval of the pre-test task error normalized with task error of the first trial).

The left plot of Figure 3 contrasts evolutional changes in normalized task errors (task errors of the 2nd–15th trials relative to the task error in the first trial) between the ER and control groups during the training sessions. For the control group, normalized task errors showed a decreasing trend with training sessions. The normalized task errors of the control group after the fourth practice trial were visibly smaller than the 95% confidence interval (CI), estimated from the task errors of the pre-test of the control group. In contrast, normalized task errors of the ER group were less susceptible to visuomotor practice. The normalized task errors of all practice sessions of the ER group were not evidently smaller than the 95% CI of pre-test errors. For the control group, the results of paired *t*-test revealed that absolute task error of the 15th trial (.694% ± .211% MVC) was significantly smaller than that of the first trial (.972% ± .359% MVC) (*t*_13_ = 5.108; *p* < .001). For the ER group, absolute task error of the 15th trial (.984% ± .346% MVC) did not significantly differ from that of the first trial (1.001% ± .316% MVC) (*t*_13_ = .243; *p* = .811). All fourteen participants (14/14) in the control group consistently exhibited reductions in absolute task errors of the last practice trial (Figure 3, the right lower plot), as compared with that in the first practice trial. In contrast, the task errors of eight of the fourteen participants (8/14) in the ER group increased in the last practice trial (Figure 3, the right upper plot).

**FIGURE 4:**
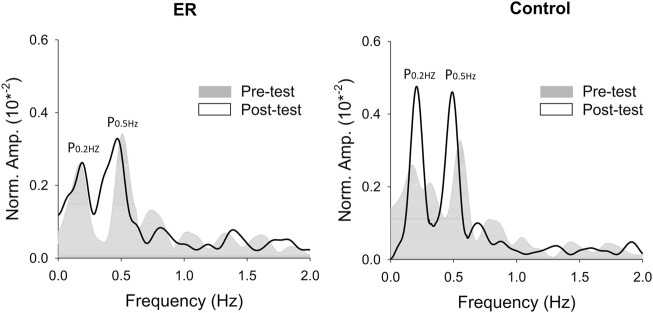
The contrast of pooled normalized power spectra of force tracking between the pre-test and post-test from representative subjects in the ER and control groups.

With respect to the training benefits, task accuracies in the time and frequency domains of the pre-test and post-test were indexed with the RMS values of the mismatches between the target and force signal (Err_RMS_) and the combined peak amplitudes of the target frequencies (P_0.2+0.5 Hz_) in the force output, respectively. Figure 4 shows a typical example of pooled power spectra of tracking force in the pre-test and post-test for the ER and control groups; Table 1 shows the results of Hotelling’s T-squared statistics, which contrast the task accuracies in the time and frequency domains between the pre-test and post-test for the ER and control groups. Task accuracies between the pre-test and post-test were significantly different in the control group (*p* = .015, *η*_*p*_^*2*^ = .505) but not in the ER group (*p* = .150). In the control group, Err_RMS_ was smaller (*p* = .002, *η*_*p*_^*2*^ = .539) and P_0.2+0.5 Hz_ (*p* = .001, *η*_*p*_^*2*^ = .555) was greater in the post-test than in the pre-test.

**TABLE 1:** Means and standard errors of task accuracies in the time/spectral domains in the pre-test and post-test for the ER and control groups. (Err_RMS_: root mean square of the task error; P_0.2+0.5Hz_: combined amplitudes of 0.2 Hz and 0.5 Hz spectral peaks in the force output).

*Task Accuracy*		Pre-test	Post-test	*Statistics*
**ER**	**Err**_**RMS**_ (% MVC)	.992 ± .247	.910 ± .265	Wilks’ Λ = .687, *p* = .150, *η*_*p*_^*2*^ = .313
	**P**_**0.2+0.5**_ ** **_**Hz**_ (*10^-2^)	.814 ± .171	.837 ± .241	
**Control**	**Err**_**RMS**_ (% MVC)	**.942 ± .307**	**.690 ± .176** **^a^**	**Wilks’ Λ = .495, *p* = .015, *η***_***p***_^***2***^ **= .505**
	**P**_**0.2+0.5**_ ** **_**Hz**_ (*10^-2^)	**.819 ± .273**	**1.041 ± .215** **^b^**	**Err**_**RMS**_ **: t**_**13**_ **=3.895, *p* = .002, *η***_***p***_^***2***^ **= .539**
				**P**_**0.2+0.5**_ **_ _**_**Hz**_ **: t**_**13**_ **= -4.023, *p* = .001, *η***_***p***_^***2***^ **= .555**

The bold values highlight values with significant differences. ^a^Post-test < Pre-test, *p* < .005.

^b^Post-test > Pre-test, *p* < .005.

### 3.2 EMG and motor units

**FIGURE 5:**
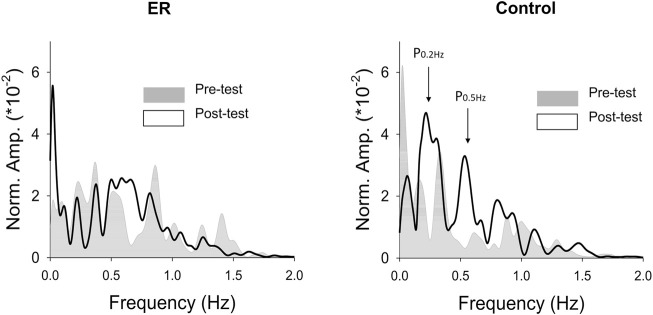
The contrast of pooled normalized power spectra of mean discharge trace (MDT) between the pre-test and post-test from representative subjects in the ER and control groups.

Across individuals, the average numbers of MUs accurately decomposed in each experimental trial in the pre-test for the ER and control groups were 23.6 ± 3.3 (range: 17.3–27.7) and 24.3 ± 4.8 (range: 18.0–29.3), respectively. In the post-test, those numbers were 26.3 ± 3.6 (range: 17.7–32.3) and 26.0 ± 3.9 (range: 17.3–30.0), respectively. Modulation in motor unit behaviors was assessed with changes in discharge patterns (M-ISI_GAV_ and CV-ISI_GAV_) of motor units and the characteristics of the mean discharge trace (RMS, P_0.2+0.5 Hz_) after training. Hotelling’s T-squared statistics were used to examine the training-related change in MU discharge pattern, and the results are summarized in Table 2(A). Only the control group showed a significant modulation of discharge variables of MUs (*p* = .003, *η*_*p*_^*2*^ = .630); the ER group did not (*p* = .294). For the control group, M-ISI_GAV_ in the post-test was greater than M-ISI_GAV_ in the pre-test (*p* = .018, *η*_*p*_^*2*^ = .360). Figure 5 shows the pooled power spectra of the pooled discharge trace in the pre-test and post-test from representative subjects in the ER and control groups, respectively. It was evident that the target frequencies (0.2 Hz and 0.5 Hz) in the pooled discharge trace of the representative subject in the control group were enhanced after training. Unlike training in the control group (*p* = .001, *η*_*p*_^*2*^ = .667), training in the ER group did not modulate the mean discharge trace (*p* = .397, *η*_*p*_^*2*^ = .143) (Table 2 (B)). Post-hoc analysis revealed that the RMS and P_0.2+0.5 Hz_ of the mean discharge trace of the control group were tuned to the training effect. Compared with those in the pre-test, RMS was smaller (*p* = .017, *η*_*p*_^*2*^ = .367) but P_0.2+0.5 Hz_ was greater (*p* = .002, *η*_*p*_^*2*^ = .550) in the post-test, respectively.

**TABLE 2:** Means and standard errors of variables of inter-spike interval (ISI) (A) and mean discharge trace (MDT) (B) in the pre-test and post-test for the ER and control groups. (M-ISI_GAV_ = the averaged value of the mean inter-spike interval for all motor units; CV-ISI: the coefficient of variation of ISI; CV-ISI_GAV_: the mean value of the coefficient of variation of inter-spike intervals for all MUs; RMS: root mean square; P_0.2+0.5Hz_: the combined amplitudes of the 0.2 Hz and 0.5 Hz spectral peaks). (A).

*ISI Variables*		Pre-test	Post-test	*Statistics*
**ER**	**M-ISI**_**GAV**_ (ms)	60.66 ± 10.12	58.54 ± 12.92	Wilks’ Λ= .815, *p* = .294, *η*_*p*_^*2*^ = .185
	**CV-ISI**_**GAV**_	.211 ± .011	.205 ± .019	
**Control**	**M-ISI**_**GAV**_ (ms)	**61.12 ± 9.34**	**64.25 ± 11.24** **^a^**	**Wilks’ Λ= .370, *p* = .003, *η***_***p***_^***2***^ **= .630**
	**CV-ISI**_**GAV**_	.216 ± .010	.214 ± .014	**M-ISI: t**_**13**_ **= -2.701, *p* = .018, *η***_***p***_^***2***^ **= .360**
				ISI-CV: t_13_ = .969, *p* = .350, *η*_*p*_^*2*^ = .067

*Mean Discharge Trace*		Pre-test	Post-test	*Statistics*
**ER**	**RMS**	.815 ± .149	.868 ± .196	Wilks’ Λ = .857, *p* = .397, *η*_*p*_^*2*^ = .143
	**P**_**0.2+0.5**_ ** **_**Hz**_	.065 ± .011	.064 ± .013	
**Control**	**RMS**	**.831 ± .151**	**.766 ± .124** **^b^**	**Wilks’ Λ = .333, *p* = .001, *η***_***p***_^***2***^ **= .667**
	**P**_**0.2+0.5**_ ** **_**Hz**_	**.057 ± .011**	**.074 ± .009** **^c^**	**RMS: t**_**13**_ **= 2.745, *p* = .017, *η***_***p***_^***2***^ **= .367**
				**P**_**0.2+0.5**_ ** **_**Hz**_ **: t**_**13**_ **= -3.988, *p* = .002, *η***_***p***_^***2***^ **= .550**

The bold values highlight values with significant differences. ^a^Post-test > Pre-test, *p* < .025.

^b^ Post-test < Pre-test, *p* < .025.

^c^ Post-test > Pre-test, *p* < .00

## 4 Discussion

Contrary to expectations, ER training on a bi-rhythmic force task did not result in significant skill improvements in the post-test. The reduction in task errors with ER was not evident in the training session. There was no evident training-related modulation of motor unit behaviors with ER. In contrast, training with feedback with no reduction in errors brought about significant task improvement, together with a greater inter-spike interval, smaller fluctuations of the pooled discharge rate, and enhanced MU discharges at the target frequencies.

### 4.1 Performance gain and force structures

It was surprising to find lack of task improvement after practice with ER for the young adults. The practice curve clearly showed a tendency of performance decline across all practice trials for the ER group, as compared with the control counterpart (Figure 3). Temporary ER-induced decline in performance was previously reported in static (Hwang et al., 2017) and rhythmic (Chen et al., 2017b) force tracking. In healthy young adults, ER feedback tended to oversimplify visuomotor control with a premature feedforward process for a false impression of task success (Chen et al., 2017b). In the initial stage of motor learning, visual error feedback can guide the subjects the way to effectively minimize committed errors by trying various movement solutions. The control group with full-size of error feedback improved their tracking performance after the third practice trial (Figure 3). However, the ER group did not reduce their task errors, except in the eighth and 10th practice trials, and their task performance had not improved at the end of the training. For the first few practice trials of the ER group, the expected beneficial effect of perceived improvement in performance might have been undermined by the destructive oversimplification strategy. This observation speaks for the existence of an intrinsic error dead-zone in the visual or sensorimotor system, as proposed by Wolpert et al. (1992). An error dead-zone indicates that error detection is a magnitude-dependent process. Only an error of at least one magnitude above the detection threshold is meaningful to a control system incorporating a feedback loop. No new corrective movements can be launched, as the control system does not respond to the range of error inputs smaller than the error dead-zone (Miall et al., 1993). However, to date, the actual size of the error dead-zone remains unclear. On average, it was at least 0.45 times the size of the execution error in the first trial. The subjects who received ER were incapable of correcting errors smaller than 0.9 times the normalized error; meanwhile, they visually perceived the execution errors at only 0.5 times their size. That is, the subjects could hardly correct the visual errors that fell into the error dead-zone (0.9*0.5 of the error size of the first trial). For the young adults, virtual perception of small errors below the dead-zone led to a false impression of task success, which led to reductions in the awareness and cognitive involvement necessary for context updating and refinement of the bi-rhythmic force tracking (Seidler et al., 2012). They prematurely finalize movement strategy without adequate corrective attempts for stratifying with the “right” performance in the latter practice trials (Chen et al., 2017b).

### 4.2 Rate coding of motor units

In line with the lack of significant practice-related changes in performance gain and force structures, ER training did not mediate MU discharge to improve their performance in the motor task in the post-test. Unlike those of the control group, motor unit behaviors did not significantly adapt to short-term ER training for mastery of the designated bi-rhythmic force task (Figures 4, 5). The skill improvement in the participants trained with the full error size was associated with the potentiated P0.2 + 0.5 Hz of the pooled discharge rate in the post-test (Table 2(B)). The scenario suggested the enhanced rate coding of motor units at the target frequency during the bi-rhythmic isometric force task (Knight and Kamen, 2007). The rhythmic discharges of motor units in unison are believed to be regulated by a neural drive of central origin (Iyer et al., 1994; De Luca et al., 2015a). Another issue pertinent to task performance is steady force control around the baseline force at 30% MVC. As force outputs are averaged twitch force of the active motor units, force variability (or unfused twitch forces) can be estimated experimentally from the size of fluctuations for summated motor unit discharge trains (Farina and Negro, 2015). Previous researches show a high resemblance between force fluctuations and cumulative series of discharge race (or MDT) (Farina and Negro, 2015; Farina et al., 2016). Based on this perspective, force variability of the baseline force can be characterized by the size of low frequency oscillations in motor unit discharge patterns under 4 Hz, or RMS value of MDT (Table 2(B)) (Vaillancourt et al., 2003; Negro et al., 2009; Castronovo et al., 2018). Unlike the control group, it is worth noting that the ER group did not demonstrate a training-related reduction in MDT size. This fact probably reflects the trained group could not add to force accuracy due to fluctuations of the baseline force level. Finally, the trained control group demonstrated a greater inter-spike interval (M_ISI_GAV_) in the post-test, rather than the ER group (Table 2(A)). This result suggested that the visuomotor training improved the efficiency of muscle activation in the control group, replicating the finding of a previous work on training-induced decline in EMG activity (Moore and Marteniuk, 1986; Lay et al., 2002; Brueckner et al., 2019). The improvement of the muscle activation efficiency can likely be attributed to a reduction in muscle coactivation for enhanced short-latency reciprocal inhibition during skill acquisition (Suzuki et al., 2012; Floeter et al., 2013). Unfortunately, ER training failed to develop effective neural strategies for a trained bi-rhythmic force task. After ER training, the young adults did not present either enhancement of coherent discharge at the target frequency or reduction in variability of motor unit discharge.

### 4.3 Methodological concerns

The primary concerns of this study were the inter-day reliability and variations in the motor units at different experimental visits, despite the electrode placement being carefully tracked across days. Several previous studies have consistently provided evidence of high intra- and inter-day absolute and relative reliability for motor unit characteristics (ICCs >0.77–0.93) with decomposition-based surface EMG across a range of submaximal contraction intensities (De Luca et al., 2015a; Martinez-Valdes et al., 2016; Colquhoun et al., 2018). Hence, motor unit variables were recommended for monitoring adaptations in a longer-term intervention study (Hoshizaki et al., 2020). In addition, the validity of EMG decomposition is another issue of debate (Farina and Enoka, 2011; De Luca et al., 2015b). We cannot completely deny the likelihood of small decomposition errors (Piotrkiewicz and Türker, 2017). To be rigorous, we applied the decompose-synthesize-decompose-compare (DSDC) test (Nawab et al., 2010; De Luca et al., 2015b) to ensure the accuracy of the decomposition results. Although the accuracy of the EMG decomposition results in this study was not independently validated, the use of the DSDC test for accuracy assessment of surface EMG decomposition was highly compatible with the results of intramuscular EMG decomposed in EMGlab software (McGill et al., 2005; Hu et al., 2014). Finally, this work did not analyze motor unit recruitment strategies between groups, acknowledging that the firing rate of a motor unit is a function of its recruitment threshold. However, this limitation is unlikely to affect the conclusions of the present work, as the force levels in both experiment groups were identical. Presumably, the recruitment thresholds of decomposed motor units were not group dependent.

## 5 Conclusion

The healthy young adults who receive error reduction feedback did not improve their performance in a rhythmic motor task after training and did not increase the modulation of their motor unit discharges at the target frequencies. In addition, visuomotor training with error reduction does not improve the efficiency of motor production for force generation, in light of the lack of adaptive reduction in the inter-spike intervals of motor units. Failure to improve visuomotor performance with error reduction associates with insignificant modulation of low-frequency neural drives to motor units. The present findings emphasize the role of sufficient visual error perception in learning to master human–machine interfaces.

## Data Availability

The original contributions presented in the study are included in the article/supplementary material, further inquiries can be directed to the corresponding author.
